# Are primary care practitioners in Barbados following diabetes guidelines? - a chart audit with comparison between public and private care sectors

**DOI:** 10.1186/1756-0500-4-199

**Published:** 2011-06-15

**Authors:** O Peter Adams, Anne O Carter

**Affiliations:** 1 Faculty of Medical Sciences, University of the West Indies, Cave Hill Campus, St. Michael, Barbados; 2 Department of Community Health and Epidemiology, Queen's University, Ontario, Canada

## Abstract

**Background:**

Over 19% of the population ≥ 40 years of age in Barbados are diabetic. The quality of diabetes primary care is uncertain.

**Findings:**

Charts of diabetic and hypertensive patients were randomly sampled at all public and 20 private sector primary care clinics. Charts of all diabetic patients ≥ 40 years of age were then selected. Processes of care, and quality targets for blood pressure (BP), fasting blood glucose (FBG) and glycosylated haemoglobin (HbA1c) were documented.

252 charts of diabetic patients (125 public and 127 private) were audited. Patients had the following characteristics: mean age 64 years, female gender 61%, mean duration of diagnosis 9 years, and hypertension diagnosed 78%. Patients had an average of 4.7 clinic visits per year, 66% were prescribed metformin, 68% a sulphonylurea, 25% a statin, 21% insulin, 15% aspirin and 12% a glucosidase inhibitor. Public patients compared to private patients were more likely to be female (77% vs. 46%, p < 0.01); have a longer duration of diagnosis (11.4 vs. 6.6 years, p < 0.01), have more clinic visits per year (5.2 vs. 4.3, p < 0.01), and to be using insulin (28 vs. 15% p = 0.01). Over a 2 year period, the proportion of charts with the following recorded at least once was: BP 98%, weight 80%, FBG 76%, total cholesterol 72%, urine tested for albumin 66%, serum creatinine 62%, dietary advice 61%, exercise advice 49%, lipid profile 48%, foot examination 41%, HbA1c 33%, dietician referral 23%, retinal examination 18%, tobacco use 17%, body mass index 0%, and waist circumference 0%. Public patients were more likely to have recorded: weight (92% vs. 68%, p = < 0.01); tests for total cholesterol (78% vs. 65%, p = 0.02), albuminuria (72% vs. 59%, p = 0.03), serum creatinine (79% vs. 44%, p < 0.01), and foot examination (50% vs. 32%, p = < 0.01); dietician referral (37% vs. 8%, p < 0.01), and tobacco use (26% vs. 8%, p < 0.01). For those tested, the most recent BP was < 140/90 for 43%, HBA1c was < 7% for 28%, and FBG was < 6.7 mmol/L for 27%.

**Conclusions:**

Interventions such as body mass assessment, lifestyle advice, screening for retinopathy, monitoring blood glucose control, and achieving BP and glycaemic targets need improvement.

## Background

Diabetes mellitus with an estimated prevalence of 19.4% in people 40 years of age and over in Barbados [1] places a significant demand on health care resources. Complications directly related to diabetes add to the problem. For example the diabetes-related lower extremity amputation rate has been estimated as 936 per 100,000 people in Barbados, making it one of the highest in the world [2]. In addition, it has been estimated that 29% of those with diabetes have diabetic retinopathy [1].

Diabetes primary care is mainly done by general practitioners. Comprehensive public sector care is done at no cost to the patient, whereas patients seen in the private sector pay a fee. Medication to treat both diabetes and hypertension is available to patients in both sectors at no cost. An audit of diabetic primary care patients conducted prior to 1995 in Barbados revealed deficiencies in care. Only 53% were controlled to a most recent fasting blood glucose (FBG) < 8 mmol/L^-1 ^or random blood glucose < 10 mmol/L^-1^, and 17% of patients on hypertension treatment had their BP controlled to < 140/90 [3].

The Commonwealth Caribbean Medical Research Council (CCMRC), now the Caribbean Health Research Council (CHRC), developed and distributed practice guidelines, *Managing Diabetes in Primary Care*, in 1995 [4]. However, aside from some seminars no specific implementation or evaluation strategies were carried out. The only published evaluation of care since the release of the 1995 CCMRC guidelines was an audit of public sector patients done some 4 months afterwards [5]. This was published in the form of a brief abstract only. In 2001 the Ministry of Health of Barbados developed the *Protocol for the Monitoring, Surveillance and Management of Diabetes Mellitus in Barbados*, which was implemented by seminars directed only at public sector health professionals [6]. A revised version of the CHRC diabetes guideline was disseminated in early 2006 [7] without any specific implementation and evaluation strategies.

Although guidelines have the potential to improve both the process of care and patient health outcomes [8,9], when they are evaluated they are often found to fall short of expectations [8,10,11]. The main reason identified for this by a systematic review was less than satisfactory adoption of guidelines by practitioners [12].

This study was done in 2005, prior to the publication and dissemination of the revised CHRC Diabetes guidelines. The aims were to evaluate the actual status of care of persons with diabetes in primary care in Barbados by means of a chart audit, to determine how closely this care adhered to the 1995 CCMRC guidelines and more recent protocol, and to provide baseline data to allow the effectiveness of the 2006 CHRC guidelines to be judged in the future. Another important aim of the clinical audit process was to identify deficiencies and provide the basis for implementing change aimed at improving patient care. A comparison of the private and public care was made as differences in system, practitioner and patient factors between sectors may affect guideline adoption and achievement of quality targets.

## Methods

### Setting

The population of Barbados was 268,792 at the last census in 2000, of which 95.6% were of African origin [13]. The island is 430 km^2^, and has a good road network. Eight public sector polyclinics strategically located around the island provide free comprehensive primary care, while in 2005 at least 89 private general practitioners were providing service for a fee. All Barbadians are entitled to public sector care. Robust data is not available but it has been estimated that primary care is approximately equally split between the public and private sectors [14].

At public sector polyclinics hypertensive patients are often seen by a nurse who may record the BP, weight and urine test results, before the consultation with the general practitioner. A dietician and podiatrist are available at each clinic on specific days. All polyclinics have a pharmacy. Most private practitioners work in solo or small group practices, and do not employ a nurse. Many patients seen privately do not have health insurance, but drugs for the treatment of diabetes and glucometer test strips are provided at no cost to both public and private sector patients by the Barbados Drug Service.

### Chart audit instrument

A chart audit instrument was developed to measure the quality of patient care using indicators derived from the Caribbean and Barbados guidelines [4,6], as well as other sources such as the Diabetes Quality Improvement Project (DQIP) [15]. The indicators used included: each visit documentation of weight, blood pressure and glucose control; biannual HbA1c; annual retinal and foot examinations, lipid profile, and screen for proteinuria/microalbuminuria; and maintenance of blood pressure below 140/90 (as recommended in the guidelines) and HBA1c below 7%. For each patient, age, sex, duration of disease, co-morbidity, medications, and health care source (private or public sector) were recorded. Identifying information concerning patients, and health care providers were not collected. Data were collected for a 2-year period ending on date of the last entry into the chart for diabetes. Processes of diabetes care were assessed by noting if they were documented at least once over a 2-year period. The 2-year interval was chosen as it allowed for reasonable delays such as for the return of lab results and delayed appointments when assessing items recommended to be done yearly.

### Chart sampling and data analysis

One hundred charts of patients with diabetes were selected from the public sector polyclinics, and the same number from 20 private physicians. Inclusion criteria were: at least one visit for diabetes in the last year by a patient at least 40 years of age; and diabetes documented in the chart at least 2 years prior to that visit. For the public sector, 100 charts were selected overall, with the number selected at each polyclinic in proportion to the number of patients seen annually by that polyclinic. Private physicians were selected from a previously validated list containing 89 names and asked to participate in a focus group study on diabetes and hypertension. At the end of the focus group session physicians were asked to participate in a chart audit. Only one refused. Nine additional physicians who could not attend focus groups because they were not available at the time the sessions were held were recruited to make a total of 20 physicians. Since the annual number of patients seen by individual private physicians is unknown, the 20 physicians were asked to contribute 5 charts each. Charts were selected randomly at the various sites, using (a) random numbers if a numerical charting system existed or (b) randomly selected shelves and files within shelves if an alphabetical system existed. This method of randomisation should allow generalisation of results to all persons over age 40 with diabetes obtaining medical care for it in Barbados in the two year period studied. An equal number of charts of patients with hypertension were also selected in the same way as part of a study on hypertension care conducted at the same time [16], and those who had diabetes were also included in this study.

### Ethical approval

Approval was obtained from the Institutional Review Board of the University of the West Indies, Cave Hill Campus and the Ministry of Health, Barbados.

## Results

A total of 253 charts of diabetic patients were audited (199 selected because the patient was diabetic and 54 because the patient was hypertensive but also had diabetes), 126 from public polyclinics and 127 from private practitioners. One public clinic contributed 2 less charts than the required number.

The mean age of patients was 64 years, the mean duration of diagnosis was 9 years, 61% were female, and 78% were hypertensive. Patients had an average of 4.7 clinic visits per year, 68% were prescribed a sulphonylurea, 66% metformin, 25% a statin, 21% insulin, 15% aspirin, and 12% a glucosidase inhibitor. Patients attending public clinics were more likely than private patients to be female, have vascular disease recorded, to be prescribed insulin, make more clinic visits per year, and to have been diagnosed with diabetes for a greater duration. Private physicians' patients were significantly heavier. Otherwise the private and public patients were similar (Table 1).

**Table 1:** Characteristics of diabetic patients according to whether they attended public or private clinics (n = 253)

Categorical Characteristic	Number (%)	Number (%) attending public clinic n = 126	Number (%) attending private clinic n = 127	P value by Chi Square	
Gender**: Male	97 (39)	29 (23)	68 (54)	< 0.01	
Female	154 (61)	97 (77)	57 (46)		
Have hypertension*	144 (72)	71 (72)	73 (73)	0.95	
Have vascular disease	33 (13)	23 (18)	10 (8)	0.01	
On Statin	63 (25)	34 (27)	29 (23)	0.45	
On Biguanide	167 (66)	77(61)	90 (71)	0.10	
On Sulphonylurea	172 (68)	80 (64)	92 (72)	0.13	
On Insulin	54 (21)	35 (28)	19 (15)	0.01	
On Glucosidase	30 (12)	12 (10)	18 (14)	0.25	
On aspirin	39 (15)	20 (16)	19 (15)	0.84	

**Continuous Characteristic**	**Number with valid data**	**Mean (SD)**	**Mean attending public clinic**	**Mean attending private clinic**	**P value by t-test**

Age (years)	252	64 (12)	65.3	63.3	.17
Weight (kg)	202	82 (21)	76.5	88.2	.00
Clinic visits/yr	251	4.7 (1.7)	5.2	4.3	< .01
Years since first recorded diagnosis	246	9.0 (5.9)	11.4	6.6	< .01

*Only the 199 persons (99 public and 100 private) selected because they were diabetic are included. The 54 persons selected because they had hypertension, but also happened to have diabetes are excluded.

** For gender, n = 251 (126 public and 125 private)

Most patients did not have a recording during a 2-year period of the following items: physical examination (height, body mass index (BMI), waist circumference, and foot and retinal examination); lifestyle history and advice (alcohol use, tobacco use, and exercise advice); laboratory investigations (HbA1c and lipid profile); referral to a dietician, and patient self blood glucose monitoring (Table 2). Public patients compared to private patients were more likely to have recorded in their charts over a 2-year period the following items: weight, foot examination, total cholesterol, serum creatinine, urine albumin, and history and/or advice on such lifestyle items as alcohol and tobacco use, diet advice as well as referral to a dietician. Urine albumin testing was recorded in 66% of the charts, with 94% of the testing being by routine clinic dipstick.

**Table 2:** Number and proportion of diabetic patients having various processes of care recorded at least once in last 2 years by health care source (n = 253 unless otherwise stated)

Process of care	Number (%) with process recorded in the chart	Number (%) attending public clinic n = 126	Number (%) attending private clinic n = 127	P value by Chi Square
Height	5 (2)	2 (2)	3 (2)	1.00
Weight	202 (80)	116 (92)	86 (68)	.00
BMI	1 (0.4)	1 (1)	0 (0)	.50
Waist circumference	0 (0)	0 (0)	0 (0)	-
Blood pressure	247 (98)	122 (97)	125 (98)	.44
Foot examination	104 (41)	63 (50)	41 (32)	.00
Retinal examination	46 (18)	24 (19)	22 (17)	.70
Fasting blood glucose	192 (76)	98 (78)	94 (74)	.51
SBGM* being done	64 (25)	29 (23)	35 (28)	.43
HbA1c†	85 (33)	48 (38)	37 (29)	.15
Urine albumin‡	165 (66)	90 (72)	75 (59)	.03
Serum creatinine	155 (62)	99 (79)	56 (44)	.00
Total cholesterol	181 (72)	98 (78)	83 (65)	.02
Lipid profile	120 (48)	63 (50)	57 (45)	.38
Alcohol use recorded	48 (19)	34 (27)	14 (11)	.00
Tobacco use recorded	42 (17)	32 (26)	10 (8)	.00
Diet advice given	153 (61)	86 (68)	67 (53)	.01
Referred to dietician	57(23)	47 (37)	10 (8)	.00
Exercise advice	124 (49)	65 (52)	59 (47)	.37

*Self blood glucose monitoring

†Glycosylated haemoglobin

‡Tests done by routine dipstick or laboratory testing.

Since weight, blood pressure, and FBG are recommended to be measured at each visit, and visits are recommended every quarter, to meet guideline recommendations, a patient should have 4 readings of each recorded per year (table 3). The remaining variables in table 3 are recommended every year, or on average, 2 in 2 years. Only the frequency of blood pressure recordings and urine dipstick testing for albumin on average met the target. Although patients of private practitioners had, on average, fewer recordings of blood pressure (also had fewer clinic visits), both settings achieved the target of 4 readings per year. Private patients had on average fewer weights, blood pressures, foot examinations, urine albumin and cholesterol tests recorded, but had more FBG tests than public patients.

**Table 3:** Means of continuous characteristics of diabetic patients according to whether they attended public or private clinic

Characteristic	Mean (SD) for all patients	Mean for public patients	Mean for private patients	P value by t test	Number with valid data
Number of diabetic medications	1.7 (0.8)	1.7	1.7	.97	252
Total number of medications	3.9 (1.8)	3.9	3.9	.96	251
Weights recorded/yr	3.0 (1.9)	3.4	2.5	.01	235
Blood pressures/yr	4.5 (1.6)	4.8	4.3	.01	251
Fasting blood glucoses/yr	1.3 (1.2)	1.1	1.6	.01	243
HbA1c/2 yr	0.7 (1.4)	0.7	0.8	.54	240
Urine albumin/2 yr	2.5 (2.8)	3.0	1.9	.00	234
Foot exams/2 yr	1.1 (1.7)	1.4	0.8	.00	245
Retinal assessments/2 yr	0.30 (0.62)	0.36	0.24	.14	247
Cholesterols/2 yr	1.1 (1.0)	1.3	1.0	.01	246

Table 4 indicates the proportion of patients meeting quality targets. Most patients had at least 4 visits per year, but low proportions met the clinical targets with the exception of diastolic blood pressure < 90 and the sub-optimal HbA1c < 10. Of persons who had tests done 27% were controlled with a FBG < 6.7, 45% had a FBG < 8 mmol/L, and 28% had an HBA1c < 7%. Despite private physicians tendency to do fewer tests and record fewer items, similar proportions of patients from the 2 sectors met clinical targets, except that private clinics exceeded public clinics in meeting the sub-optimal HbA1c target.

**Table 4:** Number and Proportion of diabetic patients meeting quality targets at last visit by health care source.

Target	Number with valid data	Number (%) meeting target	Number (%) attending public clinic who met target	Number (%) attending private clinic who met target	P value by Chi Square for difference between public and private
Systolic BP < 140	247	119 (48)	61 (50)	58 (46)	.57
Diastolic BP < 90	247	182 (74)	87 (71)	95 (76)	.40
Both systolic Bp < 140 and diastolic Bp < 90	247	106 (43)	55 (45)	51 (41)	.50
FBG* < 6.7 (good†)	192	51 (27)	31 (32)	20 (21)	.10
FBG^1 ^< 8 (acceptable†)	192	87 (45)	49 (50)	38 (40)	.18
HbA1c < 7 (optimal†)	85	24 (28)	12 (25)	12 (32)	.45
HbA1c < 10 (sub-optimal‡)	85	69 (81)	35 (73)	34 (92)	.03
4 or more visits in 1 yr	252	216 (86)	110 (88)	106 (84)	.30

*Fasting blood glucose

†As defined in Managing Diabetes in Primary Care^4 ^‡As defined in Protocol for the Monitoring, Surveillance and Management of Diabetes Mellitus in Barbados^6^

## Discussion

Despite adequate access to health care, with an average of 4.7 clinic visits per year, many patients did not have recommended processes of care such as history taking, lifestyle advice, physical examination, and laboratory tests performed, and many did not achieve blood glucose and BP control. A chart audit cannot identify many of the factors influencing quality of care. A focus group study done at about the same time as this chart audit identified barriers to good care, but also found that many practitioners were of the opinion that they provided good care [17].

### Body Mass and lifestyle modification

The control of obesity is important in the treatment of diabetes. Even though patients on average had their weight recorded 3 times per year, no chart had a recording of waist circumference, and only 1 had a BMI documented over a 2-year period. Barbadians may underestimate their level of overweight and obesity [18]. Discussing BMI and waist circumference with patients may therefore be necessary when trying to promote weight loss. Waist circumference is an independent and stronger predictor of cardiovascular risk than BMI, and is useful both in initial assessment and in monitoring the efficacy of weight loss measures [19,20]. However, waist circumference, and BMI are not mentioned in the CCMRC guideline [4], and except at initial assessment are not mentioned in the Ministry of Health protocol [6].

Documentation of dietary advice and referral to a dietician were recorded in only 61% and 23% of charts respectively with the public sector doing better than the private. A dietician is available to public patients at no cost at each polyclinic, whereas private patients are unlikely to have a dietician located in their doctor's office and in addition have to pay for this service. Women are more likely to influence eating habits in the household and attend public clinics more frequently. These facts may help to explain the higher rate of dietician referral in the public sector.

A history of alcohol and tobacco use was noted in less than 20% of charts. Despite the low prevalence of cigarette smoking in Barbados [21], this information is needed to calculate cardiac risk and to plan treatment [22].

### Eye and foot examination

Few patients had retinal examinations. Fundoscopy with adequate dilation of pupils is time consuming, and possibly the physician may lack confidence in properly performing the examination. However retinopathy is common with 29% of people with diabetes ≥ 40 years of age estimated to have the condition [1].

Foot examination was documented at least once in 2 years in only 41% of charts. Barbados has a high lower extremity amputation rate, with inadequate footwear shown to independently increase amputation risk (odds ratio 2.71, 95% confidence interval 1.23- 5.97) [2].

### Blood pressure

Most patients had hypertension, with 72% of those selected on the basis of diabetes having hypertension. Blood pressure was monitored in almost all patients and at an adequate frequency. Terminal digit preference would have affected the quality of the measurement [16]. Only 43% of all patients, including those not diagnosed with hypertension, had a blood pressure less than 140/90 mmHg at the last visit. A previous Barbados chart audit revealed a BP control rate of 17%, but included only those on treatment for hypertension in that estimate [3]. In comparison over 58% of diabetic patients receiving commercial care and Medicare in the USA in 2006 had their BP controlled to < 140/90 [23]. The UKPDS showed that 6.1 patients (95% confidence interval 2.6 to 9.5) need to be treated with tight control of blood pressure (mean blood pressure 144/82) vs. less tight control (mean blood pressure 154/87) over 10 years to prevent one patient from developing any complication, and 15.0 (12.1 to 17.9) to prevent death from a cause related to diabetes [24]. In our study 52% of our patients would have met the UKPDS tight control target.

### Glycaemic assessment

Glycaemic control was not adequately monitored, and most of those with data available were not controlled to target. In a two-year period two thirds of patients did not have an HBA1c test, 24% did not have a FBG (with an average of 1.3 tests per patient per year), and only a quarter were noted to be self blood glucose monitoring. In a majority of cases therefore neither health care worker nor patient could be confident about the level of glycaemic control attained. For the HbA1c test, important factors might be availability in the public sector (reflecting cost to the Ministry of Health), and cost to the private patient (about US$23 per test). The study was not designed to determine if physicians recognised the utility of the test. For self-monitoring, patient acceptance, and cost of the glucometer and lancets might be factors. Patient factors can be important e.g. the laboratory FBG test cannot be done unless the patient agrees to fast.

Of the patients who were monitored few attained guideline recommended glycaemic quality targets [4,6], with 28% achieving an HbA1c of < 7%, and 81% the sub-optimal target of < 10%. As few persons had this test, selection bias is likely and the findings should be cautiously interpreted. Only 45% of patients had a FBG < 8 mmol/L. This compares with a similar result found prior to the introduction of the CCMRC guidelines in 1995 in which 49% of patients attending public clinics and 66% attending private clinics had a FBG < 8 mmol/l or a random glucose < 10 mmol/l [3]. Based on voluntarily reported data by health plans in 2006, 87% of adult diabetics receiving commercial care and Medicare in the USA had HBA1c testing, > 42% had HBA1c levels < 7%, and > 70% had HBA1c levels ≤ 9% [23]. The National Health and Nutrition Examination Survey a population-based survey in the USA estimated control rates (HBA1c < 7%) for adults with a previous diagnosis of diabetes had progressively increased from 37% in 1999-2000 to 56% in 2003-2004 [25], suggesting that improvement should also be possible in Barbados.

### Laboratory tests

Forty eight percent of patients had a lipid profile in 2 years, although 72% of patients had the total cholesterol done. In the public sector the laboratory may not always have reagent to do a full profile. In the private sector the lipid profile at a cost of about US $45 may be unaffordable for the patient.

A serum creatinine was done in 44%, and urine was tested for albumin in 59% of private patients compared to 79% and 72% respectively of public patients. A creatinine plus electrolytes costs about US $45 privately. Testing for protein was mainly by dipstick for clinical proteinuria. In public clinics, a nurse tests the urine, whereas the private sector physician often does not employ a nurse. Urine can only be tested if a patient provides it, and this would be facilitated by having suitable facilities for collecting it at the clinic. Testing for microalbuminuria, which is the earliest manifestation of diabetic nephropathy, is not a requirement of the CCMRC guideline [4] but is required in the more recent Ministry of Health protocol [6].

### Treatment

The CCMRC guidelines [4] recommend that obese patients be treated with metformin, and sulphonylureas then added if necessary. More patients might therefore have been expected to have been on metformin. The significantly higher use of insulin by public compared to private patients may reflect the fact that public patients have been diagnosed with diabetes longer. Only a quarter of patients were on a statin, and 15% on aspirin. Neither of the guidelines emphasizes the use of these medications. Private patients under the age of 65 years are required to pay for statins and may be deterred by the cost.

### Gender

Private patients were twice as likely as public patients to be male. Males may be more likely to be working and have a greater income than females and therefore may be better able to afford private care, and may find attending the private practitioner less time consuming and more convenient.

### Limitations

Limitations were similar to that of the audit of hypertension care conducted at the same time [16]. As the sample included charts of patients who were hypertensive but also had diabetes, a slightly higher proportion of charts of hypertensive patients were audited than if selection was on the basis of diabetes only (72% vs. 78%).

## Conclusions

There appears to have been no progress in the quality of glycaemic control in over 10 years in Barbados despite the creation and distribution of guidelines Effective guideline adoption requires more than disseminating the guideline. Multiple ongoing interventions are needed in order to enable and reinforce the required changes, and the impact needs to be monitored and evaluated. Repeated educational sessions are needed to familiarise health care workers with guideline recommendations, and necessary resources must be provided to facilitate change. Strategies shown to be effective in some settings include repeated audit and feedback, reminder systems, patient education, academic detailing and financial incentives [12,26-28]. Altering reimbursement systems to reward practitioners who meet certain care targets would be difficult in Barbados as such information is not available to funders. Although simple office procedures such as measuring height, weight, waist circumference, and urine albumin; taking adequate histories and giving advice requires only staff time, human resources can be costly. Improving compliance with retinal examination may require the introduction of retinal photographic screening. Laboratory tests must be available in the public sector, and affordable in the private sector. In primary care many other aspects of patient care compete for physician's time as the "whole person" and not just the "diabetic" is looked after. The whole person aspects cannot be neglected, as they are likely to impact on the patient's interest and participation in their diabetic care, and adherence to the necessary regimens.

## Competing interests

The authors declare that they have no competing interests.

## Authors' contributions

OPA, AOC participated in the conception and design of the study; the acquisition, and interpretation of data; and drafting and revising the manuscript critically. AOC analysed the data. Both authors have read and approved the final manuscript.
